# Supplementary material for: CRIMP: a CRISPR/Cas9 insertional mutagenesis protocol and toolkit
Source: Nat Commun. 2024 Jun 12;15:5011. doi: 10.1038/s41467-024-49341-7 (PMC11169554; doi:10.1038/s41467-024-49341-7)
Supplement: Supplementary file 1 — Supplementary Information [file 41467_2024_49341_MOESM1_ESM.pdf]

CRIMP: a CRISPR/Cas9 Insertional Mutagenesis Protocol and Toolkit

Lee B Miles[1], Vanessa Calcinotto[1], Sara Oveissi[1], Rita J Serrano[1], Carmen Sonntag[1], Orlen Mulia[1], Clara Lee[1], and Robert J Bryson-Richardson[1*]

[1] School of Biological Sciences, Monash University, Clayton, Melbourne, VIC, 3800, Australia

[*] Corresponding author email: robert.bryson-richardson@monash.edu

Supplementary Information

Insertion line		Proportion of positive F1 embryos
Ti(actc1b^{int2}*-mTagBFP2)*	**Half body plan**	21/54 (38%)
Ti(actc1b^{int4}*-mTagBFP2-T2A-splitGFP11x7)- founder a*	**Mosaic**	7/29 (19%)
- founder b	**Half body plan**	49/158 (31%)
Ti(tdgf1^{int3}*-Gal4vp16/4xnrUAS-mTagBFP2)*	**Half body plan**	14/27 (52%)
Ti(vegfaa^{int1}*-Gal4vp16_synCoTC/4xnrUAS-mTagBFP2)*	**Mosaic**	12/51 (23.5%)

Supplementary Table 1. Founder germline transmission rates. The proportion of F1 embryos that inherited successful integration events from a founder for each target site.

Target gene-construct (Intron)	Injected	Mosaic	Half or full body plan	Total positive
**Protocol: Frozen guideRNA stock & 10 min incubation at 37°C**				
actc1b-mTagBFP2-afpUTR (Intron 2)	148	20 (13.4%)	2 (1.3%)	22 (15%)
actc1b-mTagBFP2-sGFP1-10 (Intron 2)	93	12 (12.9%)	2 (2.2%)	14 (15.1%)
actc1b-mTagBFP2-sGFP11x7 (Intron 4)	377	42 (11%)	1 (0.2%)	43 (11.4%)
**Protocol optimisation: Freshly prepared guideRNA & >30 min incubation at 37°C**				
actc1b-mTagBFP2-afpUTR (Intron 2)	126	83 (66%)	1 (1%)	84 (67%)
actc1b-mTagBFP2-sGFP11x7 (Intron 4)	115	55 (48%)	11 (10%)	66 (57%)

Supplementary Table 2. Injection efficiency of *actc1b* target sites before and after protocol optimisation. Targeting events below the dashed line are utilising the optimised protocol of freshly complexed guideRNA and an extended incubation time at 37 ^{o}C prior to injection.

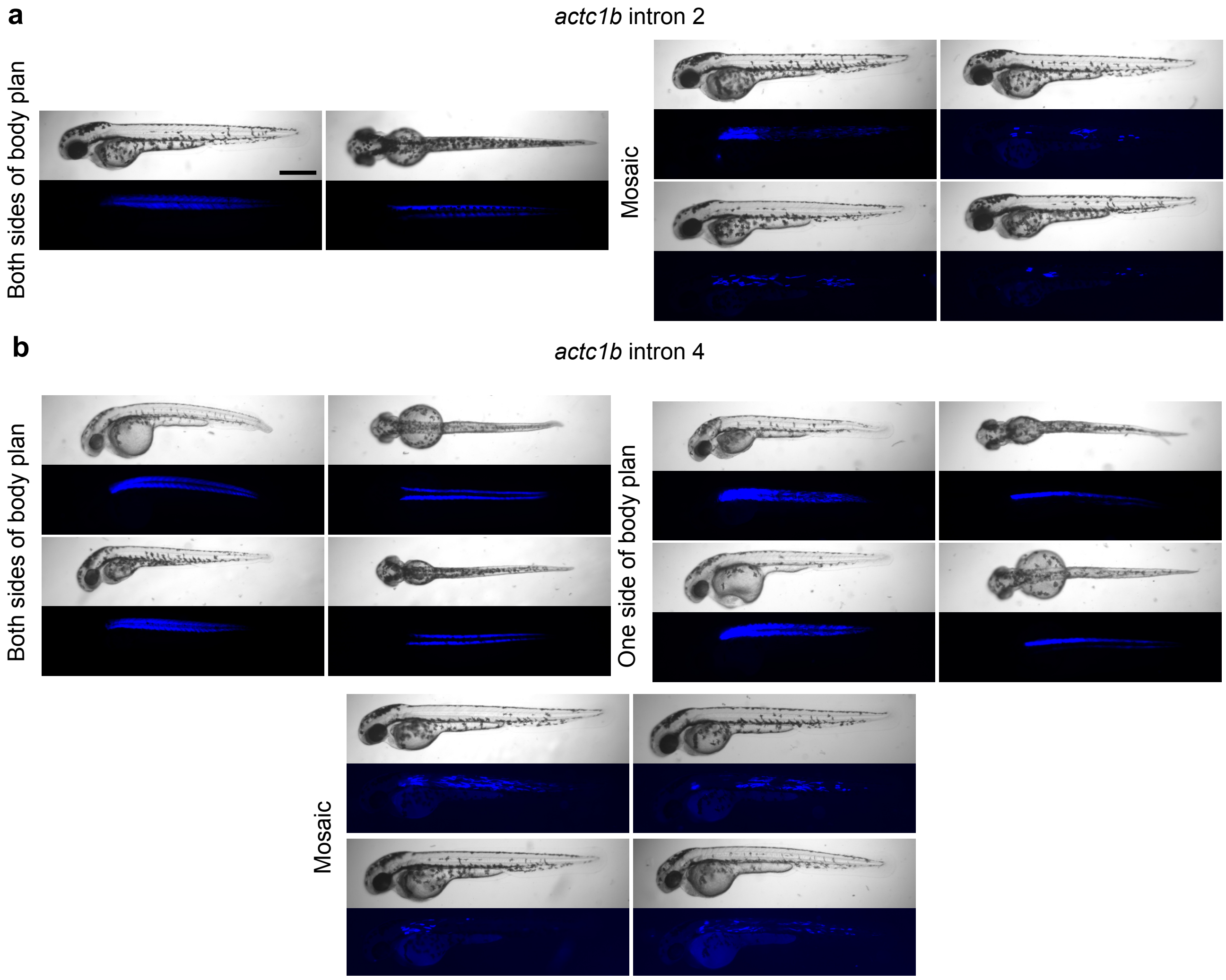

Supplementary Figure 1. Successful targeting events during the first cell division generate embryos with correct expression in one or both halves of the body plan. Optimised incubation times result in (**a**) correct integration of the targeting vector into intron-2 of *actc1b* during the first cell division as demonstrated by the embryo with *mTagBFP2* expression in both sides of the body plan. In our experience, such embryos, when raised to adulthood and crossed, have always transmitted the successful integration event to their progeny. Integration at later stages generates embryos with mosaic pattern of expression. (**b**) Integration of the targeting vector into intron-4 of *actc1b* generates embryos with mTagBFP2 expression in both sides of the body plan, as well as embryos with expression in one side of the body plan. Both expression patterns demonstrate early integration events during the first cell division. Integration events later in development result in embryos displaying *mTagBFP2* expression in a mosaic pattern. Ventral and dorsal views at 2 dpf. Scale bar indicates 500 µm.

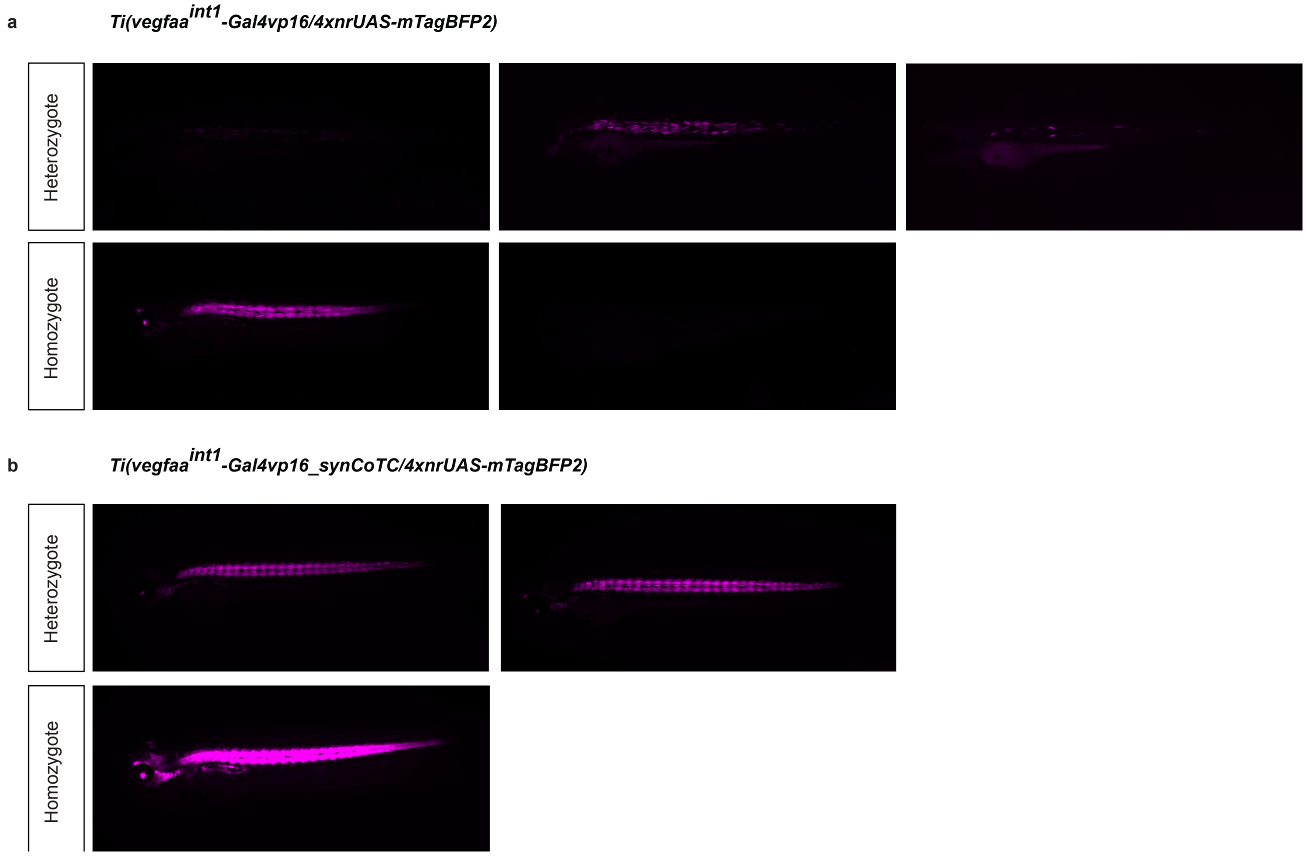

Supplementary Figure 2. Reporter expression but not phenotype is variable in the absence of a transcriptional terminator. (**a**) The *Ti(vegfaa*^{int1}*-Gal4vp16/4xnrUAS-mTagBFP2)* line that lacks a transcriptional reporter displays variation in both the level and pattern of *mTagBFP2* expression (magenta). The mutant phenotype is not affected. (**b**) Inclusion of the synCoTC terminator in the *Ti(vegfaa*^{int1}*-Gal4vp16_synCoTC/4xnrUAS-mTagBFP2)* transgenic line results in robust levels of reporter expression in all embryos without spatial variation. All images are taken at the same exposure. Experiments were repeated three independent times with similar results.

CRIMPkit collection (www.addgene.org/kits/bryson-richardson-crimpkit)

Abbreviated CRIMP plasmid name	Complete plasmid name	Addgene number	V5-tag
0_mKate2	pSA0_mKate2_synCoTC	199469	-
1_mKate2	pSA1_mKate2_synCoTC	199470	-
2_mKate2	pSA2_mKate2_synCoTC	199471	-
0_mTagBFP2	pSA0_mTagBFP2_synCoTC	199472	+
1_mTagBFP2	pSA1_mTagBFP2_synCoTC	199473	+
2_mTagBFP2	pSA2_mTagBFP2_synCoTC	199474	+
0_mTagBFP2-sGFP1	pSA0_mTagBFP2-T2A-sGFP1-10_synCoTC	199475	+
1_mTagBFP2-sGFP1	pSA1_mTagBFP2-T2A-sGFP1-10_synCoTC	199476	+
2_mTagBFP2-sGFP1	pSA2_mTagBFP2-T2A-sGFP1-10_synCoTC	199477	+
0_mTagBFP2-sGFP2	pSA0_mTagBFP2-T2A-sGFP11x7_synCoTC	199478	+
1_mTagBFP2-sGFP2	pSA1_mTagBFP2-T2A-sGFP11x7_synCoTC	199479	+
2_mTagBFP2-sGFP2	pSA2_mTagBFP2-T2A-sGFP11x7_synCoTC	199480	+
0_Gal4/UAS-mKate2	pSA0_T2A-Gal4vp16_synCoTC/4xnrUAS-mKate2	199481	-
1_Gal4/UAS-mKate2	pSA1_T2A-Gal4vp16_synCoTC/4xnrUAS-mKate2	199482	-
2_Gal4/UAS-mKate2	pSA2_T2A-Gal4vp16_synCoTC/4xnrUAS-mKate2	199483	-
0_Gal4/UAS-mTagBFP2	pSA0_T2A-Gal4vp16_synCoTC/4xnrUAS-mTagBFP2	199484	+
1_Gal4/UAS-mTagBFP2	pSA1_T2A-Gal4vp16_synCoTC/4xnrUAS-mTagBFP2	199485	+
2_Gal4/UAS-mTagBFP2	pSA2_T2A-Gal4vp16_synCoTC/4xnrUAS-mTagBFP2	199486	+
0_Gal4/UAS-mTagBFP2-sGFP1	pSA0_T2A-Gal4vp16_synCoTC/4xnrUAS-mTagBFP2-T2A-sGFP1-10	199487	+
1_Gal4/UAS-mTagBFP2-sGFP1	pSA1_T2A-Gal4vp16_synCoTC/4xnrUAS-mTagBFP2-T2A-sGFP1-10	199488	+
2_Gal4/UAS-mTagBFP2-sGFP1	pSA2_T2A-Gal4vp16_synCoTC/4xnrUAS-mTagBFP2-T2A-sGFP1-10	199489	+
0_Gal4/UAS-mTagBFP2-sGFP2	pSA0_T2A-Gal4vp16_synCoTC/4xnrUAS-mTagBFP2-T2A-sGFP11x7	199490	+
1_Gal4/UAS-mTagBFP2-sGFP2	pSA1_T2A-Gal4vp16_synCoTC/4xnrUAS-mTagBFP2-T2A-sGFP11x7	199491	+
2_Gal4/UAS-mTagBFP2-sGFP2	pSA2_T2A-Gal4vp16_synCoTC/4xnrUAS-mTagBFP2-T2A-sGFP11x7	199492	+

Supplementary Table 3. List of toolkit vectors. The presence of the V5-tag sequence in the vector is indicated with a + symbol in the V5-tag column.

Genotyping primers	Primer sequence
tdgf1_int3_F	TCTCTGGGAATGTCATGGCT
tdgf1_int3_R	AGGTGTCTAGCATTGCGGTA
β-intron_R	ACCAGCTCACCGAGAAATGA
mTagBFP_end_F	AGCTGGGACACAAGCTGAAT
vegfaa_int2_F2	TCTGTAGGCGGGAAAGAAGA
vegfaa_int2_R4	AGCCAAATATTGTCCTAACAAAGTG
T2A_gal4_qPCR_R	GAAGTTTCATTGGGCCAGGG
bag3_int3_F	TGAATGAGCAAGGGTTTCTGC
bag3_ex3_R	GGGCACTTGTTCACGGTAGA

qRT-PCR primers	
vegfaa_qPCR_F	CCCACGATATCACACTCGGT
vegfaa_qPCR_R	GGATGTACGTGTGCTCGATCT
vegfab_qPCR_F	GGTGCTGCAATGATGAAATG
vegfab_qPCR_R	TGTCACCCTGATGACGAAGA
rpl13_qPCR_F	TAAGGACGGAGTGAACAACCA
rpl13_qPCR_R	CTTACGTCTGCGGATCTTTCTG
EF1α_F	CTGGAGGCCAGCTCAAACAT
EF1α_R	ATCAAGAAGAGTAGTACCGCTAGCATTAC
mTagBFP2_R	GGTTGTCCACAGTTCCCTCC
V5_R	GGGTTTGGGATTGGCTTTCC
UBC _F	AGCTCCTCCACACGAATTC

crRNA guide sequence(PAM)		On-target score	Off-target score
tdgf1_int3	TGTTACATTGGTTGATAATA(GGG)	73	42
vegfaa_int1	ATACTAATATAGGACCACCC(AGG)	91	94
actc1b_int2	TAGATTTAGGACAAGTCTGC(TGG)	87	79
actc1b_int4	ATCTACTTAGACCTCACATG(TGG)	79	92
bag3_int2	TCCTCACACAAAAGAGCCTG(CGG)	63	78
Hbait	GAAGATCGGCCACTACATTC(TGG)		

Supplementary Table 4. Primer and guideRNA sequences. Genotyping primers, qRT-PCR primers, and guide site sequences using in this study. GuideRNA PAM sequence is indicated by brackets. IDT predicted on-target and off-target scores are provided for each guide site.

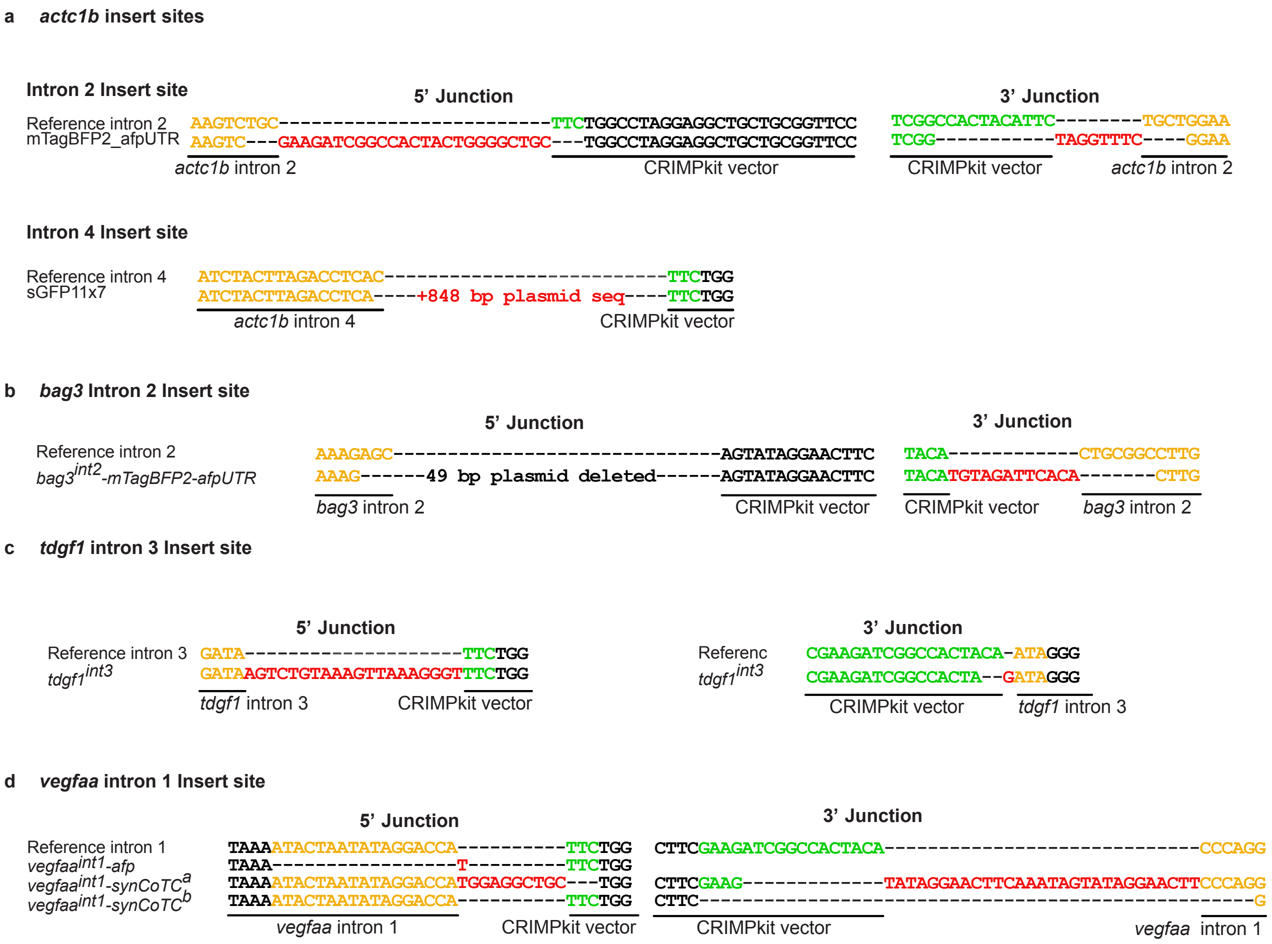

Supplementary Figure 3. Insert site sequences for targeted integrations (**a**) Sequence of the insertion site for the *actc1b*. Multiple copies of the plasmid in the target site prevented insert site sequencing in the sGFP1-10 line. *actc1b* guideRNA sequence is coloured yellow. (**b**) Sequences of the *bag3* insertion sites. *bag3* guideRNA sequence is coloured yellow. (**c**) Sequences of the *tdgf1* insertion sites. *tdgf1* guideRNA sequence is coloured yellow. (**d**) Sequences of the *vegfaa* insertion sites. *vegfaa* guideRNA sequence is coloured yellow. Hbait guideRNA sequence coloured green. Red indicates extra bases added during insertion.

CRIMP Injection protocol

1 Resuspend Alt-R crRNA and tracrRNA in Nuclease-Free IDTE Buffer to final stock concentrations of 100 µM each

- 2 nM = 20 µl
- 5 nM = 50 µl

This is stored in the -20°C freezer once resuspended

2 Make guideRNA complex

Make fresh gRNA complexes for each injection
Note: you will need 1 µl genomic guideRNA and 0.5 µl Hbait (plasmid) guideRNA per injection

2.1 Mix the following components to create a 30 µM gRNA solution: - only make enough for your injection.

Component	Pick the most suitable volumes for your requirements	
100 µM Alt-R™ CRISPR-Cas9 crRNA	0.25 µl	0.4 µl
100 µM Alt-R™ CRISPR-Cas9 tracrRNA	0.25 µl	0.4 µl
Nuclease-Free Duplex Buffer (IDT)	0.33 µl	0.53 µl
Final volume	0.83 µl	1.33 µl

2.2 Heat at 95°C for 5 min (in PCR machine).

2.3 Remove from heat, and allow to cool to room temperature (15–25°C) on your bench top.

Note: the final concentration for the crRNA is 360 ng/µl and for the tracrRNA is 670 ng/µl.

Therefore total guideRNA complex concentration is 1030 ng/µl (30 µM = 1030 ng/µl).

Note2: this complex can be stored at -20°C for up to three months with no loss of activity (when at least 30 µM), but is best to make fresh.

3 Assemble the mix (CRISPR/Cas9 complexes) for injection:

Component	Amount	Final concentration
1M KCL (0.2 µm filter sterilised) [1]	1.5 µl	300 mM
genomic guideRNA complex (from step 2.3) @1030 ng/µl	1µl	206 ng/µl (pg/µl)[2]
Hbait plasmid guideRNA complex (from step 2.3) @1030 ng/µl	0.5 µl	103 ng/µl (pg/nl)[2]
Targeting plasmid -> 36 fmol total plasmid [3] (dilute so 0.5 µl is sufficient)	0.5 µl	7.2 fmol/µl
phenol red (0.05%) (0.2 µm filter sterilised)	0.5 µl	0.005%
H20 (sterile MilliQ)	to final volume 5µl	
Cas9 HiFi V3 protein (IDT™) (62 µM = 10,000 ng/µl)	0.3 µl	700 ng/µl (pg/nl)
Final volume	5µl	

[1] Required for solubility of complex - see Burger et al. (2016)
[2] See Shah et al. (2015) describing non-lethal injection of high amounts of guideRNA
[3] Calculate plasmid fmoles at NebBioCalculator https://nebiocalculator.neb.com/#!/dsdnaamt

4 Incubate at 37°C for >30 min before injecting into one-cell stage embryos

5 Inject embryos with CRISRP/Cas9 Ribonuclear (RNP) complex

5.1 Collect embryos within 5 minutes of being laid and inject 2 nL of CRISRP/Cas9 Ribonuclear (RNP) complex directly into the cell of the embryo.

Note: In our experience injecting into the embryos within the first 15 minutes increases the likelihood of integration before (or during) the first cell division, and maximised the chance of obtaining embryos with reporter expression in half of the embryo body plan.

5.2 Incubate injected embryos at 28 °C and remove any unfertilised or dead embryos before leaving for the day.

5.3 Screen injected embryos for positive reporter expression at 24 hpf (or a time point after target gene is expressed).

References

Burger, A., Lindsay, H., Felker, A., Hess, C., Anders, C., Chiavacci, E., Zaugg, J., Weber, L. M., Catena, R., Jinek, M., Robinson, M. D., and Mosimann, C. Maximizing mutagenesis with solubilized CRISPR-Cas9 ribonucleoprotein complexes. *Development*, 143(11):2025–2037, Jun 2016. [DOI:10.1242/dev.134809] [PubMed:27130213].

Shah, A. N., Davey, C. F., Whitebirch, A. C., Miller, A. C., and Moens, C. B. Rapid reverse genetic screening using CRISPR in zebrafish. *Nat Methods*, 12(6):535–540, Jun 2015. [PubMed Central:PMC4667794] [DOI:10.1038/nmeth.3360] [PubMed:25867848].

Supplementary Figure 4: CRIMP Injection Protocol. A summary of the protocol for lab use.

Supplementary References

1 Burger, A. *et al.* Maximizing mutagenesis with solubilized CRISPR-Cas9 ribonucleoprotein complexes. *Development (Cambridge, England)* **143**, 2025-2037, doi:10.1242/dev.134809 (2016).

2 Shah, A. N., Davey, C. F., Whitebirch, A. C., Miller, A. C. & Moens, C. B. Rapid reverse genetic screening using CRISPR in zebrafish. *Nature Methods* **12**, 535-540, doi:10.1038/nmeth.3360 (2015).
