# Supplementary material for: CRIMP: a CRISPR/Cas9 insertional mutagenesis protocol and toolkit
Source: Nat Commun. 2024 Jun 12;15:5011. doi: 10.1038/s41467-024-49341-7 (PMC11169554; doi:10.1038/s41467-024-49341-7)
Supplement: Supplementary file 3 — Reporting Summary [file 41467_2024_49341_MOESM3_ESM.pdf]

nature portfolio

Corresponding author(s): Robert Bryson-Richardson

Last updated by author(s): May 8, 2024

Reporting Summary

Nature Portfolio wishes to improve the reproducibility of the work that we publish. This form provides structure for consistency and transparency in reporting. For further information on Nature Portfolio policies, see our Editorial Policies and the Editorial Policy Checklist.

Statistics

For all statistical analyses, confirm that the following items are present in the figure legend, table legend, main text, or Methods section.

n/a	Confirmed	
☐	☒	The exact sample size (n) for each experimental group/condition, given as a discrete number and unit of measurement
☐	☒	A statement on whether measurements were taken from distinct samples or whether the same sample was measured repeatedly
☐	☒	The statistical test(s) used AND whether they are one- or two-sided<br>*Only common tests should be described solely by name; describe more complex techniques in the Methods section.*
☒	☐	A description of all covariates tested
☒	☐	A description of any assumptions or corrections, such as tests of normality and adjustment for multiple comparisons
☒	☐	A full description of the statistical parameters including central tendency (e.g. means) or other basic estimates (e.g. regression coefficient) AND variation (e.g. standard deviation) or associated estimates of uncertainty (e.g. confidence intervals)
☐	☒	For null hypothesis testing, the test statistic (e.g. F, t, r) with confidence intervals, effect sizes, degrees of freedom and P value noted<br>*Give P values as exact values whenever suitable.*
☒	☐	For Bayesian analysis, information on the choice of priors and Markov chain Monte Carlo settings
☒	☐	For hierarchical and complex designs, identification of the appropriate level for tests and full reporting of outcomes
☒	☐	Estimates of effect sizes (e.g. Cohen's d, Pearson's r), indicating how they were calculated

Our web collection on statistics for biologists contains articles on many of the points above.

Software and code

Policy information about availability of computer code

Data collection	pco.camware V04.11/1527/64bit, ThorImageLS v4.0.2019.8191,LightCycler480 v1.5.1.62
Data analysis	Excel 16.32 (OSX), GraphPad Prism 9.3.1.350, ImageJ2 2.9.0,1.53t, A Plasmid Editor (ApE) v3.0.9, CodonZ (Horstick, E. J. et al. Increased functional protein expression using nucleotide sequence features enriched in highly expressed genes in zebrafish. Nucleic acids research 43, (2015)).

For manuscripts utilizing custom algorithms or software that are central to the research but not yet described in published literature, software must be made available to editors and reviewers. We strongly encourage code deposition in a community repository (e.g. GitHub). See the Nature Portfolio guidelines for submitting code & software for further information.

Data

Policy information about availability of data

All manuscripts must include a data availability statement. This statement should provide the following information, where applicable:

- Accession codes, unique identifiers, or web links for publicly available datasets
- A description of any restrictions on data availability
- For clinical datasets or third party data, please ensure that the statement adheres to our policy

Source data are provided with this paper. CRIMPkit vectors are available from Addgene as a complete kit (#1000000225) or individual plasmids (199469-199492).

The CRIMPkit vector sequence data has been deposited in GenBank database under accession numbers: PP297139-PP297162. The raw data and statistical analysis files used in this study are available in the Monash University Bridges data repository https://doi.org/10.26180/c.7217754.v5.

Research involving human participants, their data, or biological material

Policy information about studies with human participants or human data. See also policy information about sex, gender (identity/presentation), and sexual orientation and race, ethnicity and racism.

Reporting on sex and gender	N/A
Reporting on race, ethnicity, or other socially relevant groupings	N/A
Population characteristics	N/A
Recruitment	N/A
Ethics oversight	N/A

Note that full information on the approval of the study protocol must also be provided in the manuscript.

Field-specific reporting

Please select the one below that is the best fit for your research. If you are not sure, read the appropriate sections before making your selection.

☒ Life sciences ☐ Behavioural & social sciences ☐ Ecological, evolutionary & environmental sciences

For a reference copy of the document with all sections, see nature.com/documents/nr-reporting-summary-flat.pdf

Life sciences study design

All studies must disclose on these points even when the disclosure is negative.

Sample size	For experiments with mutant fish, the number of embryos varied according to the size of the spawn. For conditions that replicates were used, 96 larvae for each condition were collected and genotyped to identify a minimum of 14 homozygote mutants. The number of identified homozygote embryos determined the number of samples for all other genotypes.
Data exclusions	No data was excluded
Replication	Experiments were repeated three independent times, using either three or four independent biological replicates. All attempts at replication were successful.
Randomization	Embryos were randomly assigned
Blinding	Where mutant fish were compared to WT siblings, samples were blinded to the investigator and quantification and analysis was performed prior to genotyping.

Reporting for specific materials, systems and methods

We require information from authors about some types of materials, experimental systems and methods used in many studies. Here, indicate whether each material, system or method listed is relevant to your study. If you are not sure if a list item applies to your research, read the appropriate section before selecting a response.

Materials & experimental systems

n/a	Involved in the study	
☐	☒	Antibodies
☒	☐	Eukaryotic cell lines
☒	☐	Palaeontology and archaeology
☐	☒	Animals and other organisms
☒	☐	Clinical data
☒	☐	Dual use research of concern
☒	☐	Plants

Methods

n/a	Involved in the study	
☒	☐	ChIP-seq
☒	☐	Flow cytometry
☒	☐	MRI-based neuroimaging

Antibodies

Antibodies used	mouse anti-myosin heavy chain (DSHB Cat# A4.1025, RRID:AB_528356), Goat anti-mouse IgG (H+L) Alexa Flour 488 (Thermo Fisher Scientific, Cat# A-11001, RRID AB_2534069
Validation	A4.1025 was initially applied to zebrafish by Bladen et al., 1997. They demonstrated it recognised a protein of expected molecular weight in zebrafish and recognised bovine cardiac myosin. Myosin being very highly conserved between species. they also demonstrated it labeled the muscle tissue in the zebrafish.<br>Blagden, C. S., Currie, P. D., Ingham, P. W. & Hughes, S. M. Notochord induction of zebrafish slow muscle mediated by Sonic hedgehog. Gene Dev 11, 2163-2175 (1997).

Animals and other research organisms

Policy information about studies involving animals; ARRIVE guidelines recommended for reporting animal research, and Sex and Gender in Research

Laboratory animals	Zebrafish (Danio rario), TU/TL background, were used in this study. Phenotypes were examined during embryo stages and the ages reported for each figure.
Wild animals	The study did not involve the use of wild animals
Reporting on sex	Phenotypes were examined before sex is determined and so sex was not reported
Field-collected samples	The study did involve samples collected from the field
Ethics oversight	This study was approved by the School of Monash University Animal Ethics Committees (ERM18912, 21168).

Note that full information on the approval of the study protocol must also be provided in the manuscript.

Plants

Seed stocks	N/A
Novel plant genotypes	N/A
Authentication	N/A
